# Reimagining the approach for advancing maternal health equity through authentic patient engagement and research practices

**DOI:** 10.3389/frhs.2024.1474149

**Published:** 2024-11-20

**Authors:** Karey M. Sutton, Shelby Wyand, Chandra A. Char, Asli McCullers

**Affiliations:** ^1^ Center for Health Equity Research, MedStar Health Research Institute, Columbia, MD, United States; ^2^ Department of Family Medicine, Georgetown University Medical Center, Washington, DC, United States

**Keywords:** maternal health, health equity, health disparity, patient engagement, research

## Abstract

High maternal mortality and morbidity rates continue to significantly impact the United States, with Black birthing individuals being two to three times more likely to die from pregnancy related causes compared to other races. Ongoing discussions are crucial to improving care delivery and amplifying the experiences and needs of marginalized survivors of pregnancy-related psychological harm. Thus, this commentary leverages current literature and vignettes to deliver recommendations on authentically engaging patients in the cross-sectoral process of dismantling harmful clinical and research practices, thus building a safe, equitable future for maternal health.

## Introduction

The United States (U.S.) faces a profound maternal health crisis, starkly illustrated by its disproportionately high Maternal Mortality Rates (MMRs), mainly affecting Black birthing individuals. Despite being a developed nation, the U.S. maternal mortality rate is the highest among its peers, exceeding that of countries like the United Kingdom, Australia, Spain, and Germany by a significant margin (1). In June 2022, The White House released a Blueprint for Addressing the Maternal Health Crisis, highlighting these alarming statistics and calling for urgent action to address systemic disparities (2).

A Centers for Disease Control and Prevention (CDC) study reported that Black birthing individuals were three times more likely than their white counterparts to experience maternal harm (3). This disparity widens with age, where Black-birthing individuals over 30 years old face four to five times higher mortality rates compared to white-birthing individuals (3). Moreover, MMRs have increased, and disparities have widened from 2018 to 2021 (4). A study analyzing vital registration and census data from across the U.S. revealed higher MMRs in 2019 among American Indian or Alaska Native (from 14.0 to 49.2 per 100,000 live births) and Black populations (from 26.7 to 55.4 per 100,000 live births) compared to Asian, Native Hawaiian, or Other Pacific Islander (from 9.6 to 20.9 per 100,000 live births), Hispanic (from 9.6 to 19.1 per 100,000 live births), and White populations (from 9.4 to 26.3 per 100,000 live births) (5).

Similarly, Severe Maternal Morbidity (SMM), including cardiovascular concerns, diabetes, bleeding, anemia, and depression and anxiety, follow similar trends as maternal mortality (6). A retrospective study examining hospital admissions in the United States from 2002 to 2014 revealed that Black birthing individuals in the United States have the highest proportion of SMM across all pregnancy intervals, experiencing a 70% greater risk of SMM during the antepartum period compared to white birthing individuals. In contrast, Hispanic birthing individuals exhibit a 19% lower risk during the postpartum period. These findings highlight varying racial and ethnic disparities in SMM types and timing, underscoring the need for targeted interventions and policies (7). In addition, a multistate retrospective cohort study analyzing data from 2015 to 2020 indicated that the rate of SMM has increased across all racial and ethnic groups, with disparities persisting and even widening (8). Black race, Hispanic ethnicity, and delivery at Black-serving delivery units independently and interactively increased the adjusted odds of experiencing SMM (8).

Disparities in SMM and MMR persist regardless of socioeconomic status, challenging the assumption that higher education or income levels provide protective benefits. A 2019 CDC report analyzing data from 2007 to 2016 found that college-educated Black birthing individuals faced a maternal mortality rate 5.2 times higher than their white counterparts of similar educational attainment (3). Additionally, a study in California found that Black mothers in the highest income brackets were twice as likely to die from childbirth than their White counterparts (9). These findings provide evidence that assumed “equalizing” factors such as income or socioeconomic fail to buffer the impact of racism, implicit bias, and other forms of injustice on maternal outcomes for Black birthing people.

Approximately 60% of maternal deaths and nearly 90% of SMM events are preventable (6). A national retrospective review found that the most frequent preventable factors of maternal morbidity were provider-related and/or system-related, including inadequately trained providers, misdiagnosis or failure to recognize high-risk status, and untimely or unsuitable treatment (10). These provider-related and system-related factors disproportionately affected Black birthing individuals. Given these realities—high rates of maternal harm, significantly elevated risks among Black birthing individuals, the preventability of most maternal harm incidents, and the frequent involvement of healthcare providers and systems—the issue transforms into a critical patient safety crisis. More precisely, it manifests as a maternal safety crisis that disproportionately impacts Black birthing individuals.

While morbidity and mortality rates capture part of the picture, they fail to encompass the enduring effects of maternal harm, which can significantly impact birthing individuals' future family planning, overall health, and ability to care for their families (10). Qualitative research has sought to amplify the mental health burden of maternal harm, with survivors of birthing trauma sharing testimonies about emotional wounds, fear, loneliness, disrupted relationships, and detachment from their infant (11–13). Media coverage, social media discussions, and documentaries frequently highlight maternal health disparities, further intensifying fear among Black birthing individuals. For example, the Hulu documentary “Aftershock,” which highlights gripping stories of Black maternal mortality, makes information on health inequities and adverse birth outcomes more accessible to Black birthing individuals and may influence their perspectives on childbearing (14). Moreover, a Washington Post article entitled “For some Black birthing individuals, the fear of death shadows the joy of birth” highlights how awareness of these disparities overshadows what should be a joyful maternal experience (15). Findings from the Postpartum Mothers Mobile Study reveal that chronic concerns about racial discrimination in healthcare contribute to significant disparities in preterm birth rates between Black and White mothers (16). This underscores that racial discrimination within the healthcare system is not just a theoretical concern but a tangible source of stress for Black mothers, affecting their maternal health experiences.

For instance, meet Janine, who was pregnant with her first child and was admitted to the hospital due to experiencing symptoms of elevated blood pressure. During her hospital stay, Janine faced microaggressions, including assumptions that she was a single mother despite her husband Charles being present, unsolicited education about public benefits, and dismissal of her requests for information about her medication. Janine and Charles felt that the care she received was substandard and felt mistreated throughout the delivery of their baby boy. Although this was supposed to be the most exciting time for Janine and Charles, it turned out to be one of the most horrific experiences that still haunted them and affected their decision to have more children. Many stories similar to Janine's have occurred to Black birthing individuals and are independent of education and income level. Research on the psychological impact of traumatic perinatal experiences on birthing individuals and their families remains limited.

## Discussion

Effectively addressing maternal psychological harm requires a holistic approach that integrates patient experience and perspectives into patient safety frameworks. While there are emerging frameworks that specifically provide strategies for improving maternal safety (17, 18), true equity in maternal health outcomes requires an “all hands” approach to maternal safety that employs not only maternal health leaders but also leaders across the spectrum of care provision, including health equity, mental health, and patient safety experts and advocates. Also, explicitly naming racism as a core determinant of maternal disparities should be regarded as a key priority in dismantling maternal inequities with a historically informed lens. Remedying the longstanding disenfranchisement, oppression, and injustice faced by communities harmed by structural racism goes beyond “acknowledgment.” This work should extend to the confrontation of racist practices, such as implicit bias, that keep Black birthing individuals at disproportionate risk of maternal harm. Finally, recognizing and mitigating the psychological toll of racial discrimination and unequal treatment of Black birthing individuals within healthcare settings is essential for improving maternal health outcomes. Therefore, our call to action for health service researchers is to center research on the lived experiences of patients and communities to understand the role of psychological harm. Approaches such as Community-Based Participatory Research (CBPR) exemplify how to engage patients and communities as equal partners, ensuring that research addresses their needs and priorities. Authentic engagement requires valuing and compensating patients for their expertise (19). Thoughtful integration of patient perspectives and experience is needed in (1) design, (2) process, (3) analysis, (4) implementation, and (5) evaluation. Models like Culturally Responsive and Equitable Evaluation (CREE) emphasize the importance of ongoing reflection and adaptation, ensuring research remains inclusive, culturally relevant, and responsive to patient feedback (20).

Incorporating mixed methods research—combining qualitative and quantitative approaches—advances understanding of the depth of patient experiences while also providing measurable evidence that can inform practice and policy. Mixed methods can reveal what is happening and why, offering a more comprehensive understanding of patient needs. Additionally, applying an intersectional lens is crucial, as it considers how overlapping identities such as race, gender, socioeconomic status, and disability shape patient experiences (21). This approach ensures that research captures the complexities of patients' lives, leading to more targeted and effective interventions.

Additionally, acknowledging and respecting the autonomy of birthing individuals is crucial for effective patient engagement in maternal health. Co-creating birthing plans is an effective strategy to enhance this engagement by aligning patient preferences with the care they receive. It is important to recognize that the successful implementation of birthing plans requires the active participation of patients and the support and respect of maternity healthcare professionals. These plans should be integrated into the care process in a way that reflects the individual's preferences and values while also considering the perspectives and expertise of the healthcare team. Ensuring that birthing plans are honored and adapted as needed throughout the care journey promotes a collaborative and respectful approach to maternal health care (22). Another tool to engage patients in maternal healthcare is text messaging to track depression and anxiety symptoms among birthing individuals (23). Though the approach could vary for different communities and patient populations, patient engagement and shared decision-making are central to the mission (24). It is important also to consider the broader factors, such as the availability of healthcare resources and the systemic barriers that affect access to care. By addressing these more significant issues, healthcare interventions can move beyond individual-level approaches, avoiding narratives that place undue blame on birthing individuals. This shift supports a more comprehensive strategy that recognizes the impact of social determinants of health on maternal mental health outcomes. Overall, these patient engagement strategies to advance maternal health equity (Table 1) include (1) a personalized approach- meeting birthing individuals where they are, (2) recognizing social drivers for care equity, (3) addressing holistic health, (4) clarifying and softening the experience of healthcare and, (5) instilling a sense of humility through warmth, connection, and representation (25).

**Table 1:** Recommendations for action: amplifying voices in maternal health.

Recommend actions	For researchers	For healthcare facilities
1. Implement Authentic Patient Engagement and Shared Leadership * Compensation for patients and community members for their time and insights is critical	• Engage birthing individuals as co-leaders in research and safety initiatives. • Use focus groups, community advisory boards, and patient councils to gather insights and inform research. • Apply frameworks like Community-Based Participatory Research (CBPR) (14) or Patient-Centered Outcomes Research (PCOR) to ensure research addresses community needs (34).	• Establish patient advisory boards and integrate patient feedback directly into care protocols. • Train staff in active listening and culturally sensitive communication to ensure patient feedback leads to tangible changes in care delivery. • Implement shared decision-making models and collaborative care teams in healthcare systems
3. Enhance Communication and Trust-Building	• Study effective communication strategies that improve patient-provider relationships. • Develop interventions that address implicit biases and microaggressions.	• Foster a culture of trust by employing respectful communication strategies, affirming and culturally sensitive. • Use practices that affirm diverse experiences, such as active listening, maintaining eye contact, respectful acknowledgment of patient concerns, and family involvement in care discussions • Implement ongoing provider training to address implicit biases and microaggressions, ensuring equitable and empathetic care delivery.
3. Implementation of Tailored Birthing Plans	• Research best practices for co-creating birthing plans that align with cultural and individual needs. • Study barriers to implementing personalized birthing plans and explore strategies to enhance provider adherence to patient preferences.	• Encourage co-creating birthing plans that reflect individual values, cultural contexts, and medical needs. • Train healthcare providers to respect and adapt these plans throughout the care journey, upholding patient autonomy and promoting collaborative care.
5. Leverage Technology for Engagement	• Develop and evaluate digital tools, such as mobile apps and text messaging platforms, for tracking symptoms and collecting patient feedback. • Assess the effectiveness of these tools in improving patient-provider communication and care outcomes.	• Utilize technology to maintain ongoing patient communication, provide support, and gather real-time feedback on their experiences.
6. Monitor, Evaluate, and Measure Engagement Outcomes	• Routinely assess the impact of patient engagement strategies on maternal health outcomes using Culturally Responsive and Equitable Evaluation (CREE) principles (20). • Collect qualitative and quantitative data on how patient voices influence care quality and equity.	

Strategies such as meaningful patient engagement have proven influential in personal health decisions and scientific research, policy, and healthcare more broadly (26). This includes but is not limited to respecting and acknowledging the patient's perspective and using encouraging and affirming practices (27). Such practices include maintaining eye contact during patient interactions, acknowledging family members in attendance with the patient, and upholding strong empathy skills regarding the experiences and needs of patients of culturally diverse backgrounds (27). Previous research defines patient engagement as a collaborative effort involving patients, family members, and healthcare providers, where patients and their families actively participate in the healthcare team (22). The experiences of patients and their families play a crucial role in shaping healthcare delivery, and recognizing the diversity of these experiences is essential (28, 29). Not all birthing individuals share the same experiences, highlighting the need for personalized and inclusive approaches to care. Engaging patients and community members throughout the research process, from conceptualization to implementation, has become increasingly valued in healthcare settings, emphasizing their role as critical partners in improving health outcomes (30). However, the actual impact of patient engagement is not well known (30). Utilizing the AAMC Principles of Trustworthiness (31), we offer recommendations for incorporating and amplifying voices of birthing individuals into patient practice and safety. As scientists and clinicians, we are not the only experts. It is equally important for healthcare organizations to deem themselves worthy of the trust of birthing persons through intentional engagement and displays of humility.

We recognize that addressing challenges related to health disparities requires a multi-pronged approach, including national, regional, and individual-level solutions (32). Part of this approach is a nationwide commitment to ensure healthcare equitably through health insurance coverage, resource investment, public and clinical accountability, and adequately provisioned quality improvement strategies that engage patients (32). Additionally, we must develop and maintain community-based partnerships. The emphasis on relationships between healthcare staff, patients, and community-based partners can enhance the healthcare experiences of birthing individuals (33).

### Amplifying voices in maternal health

**Figure 1:**
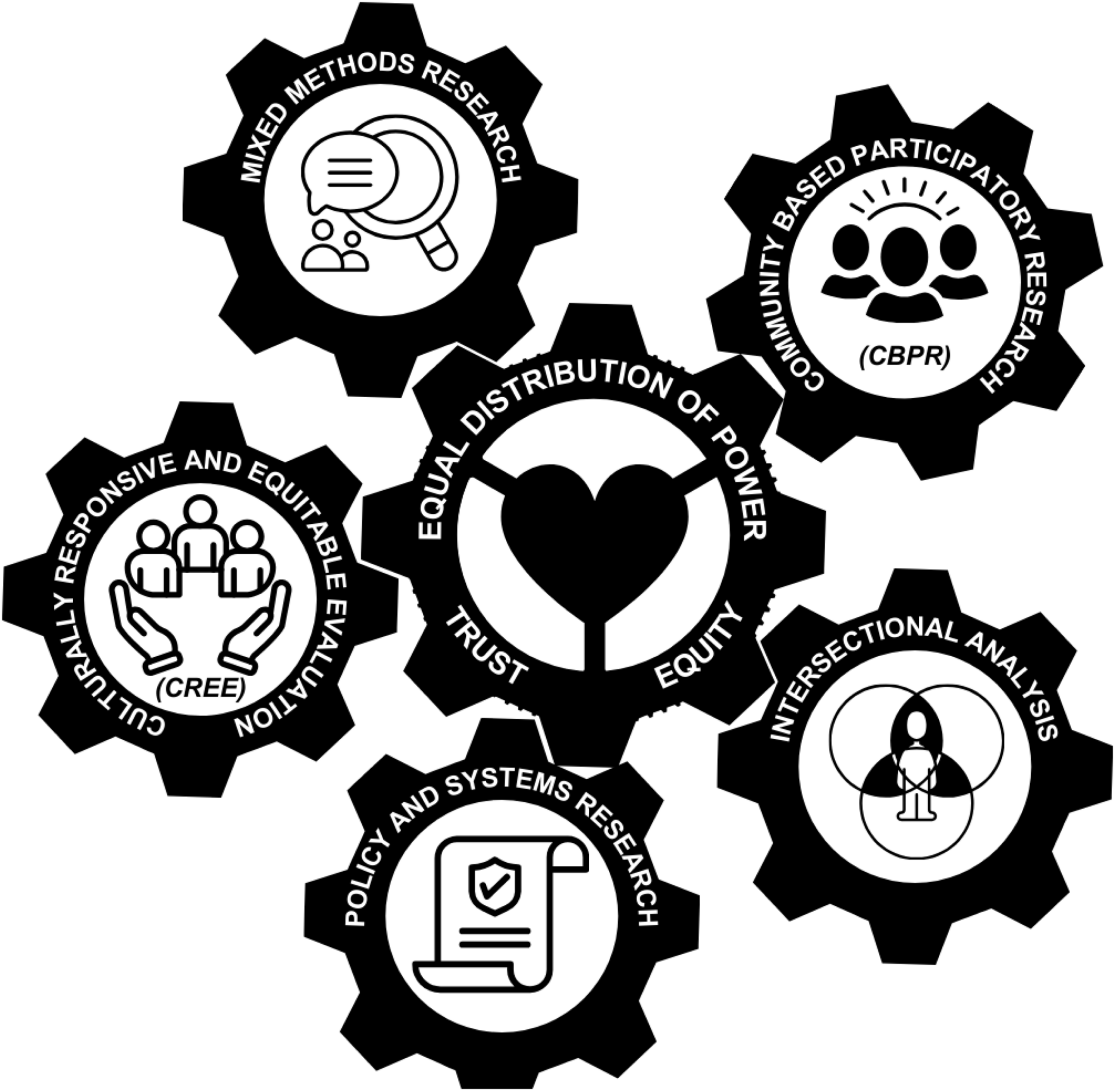
Conceptual framework for advancing maternal health research.

Figure 1 presents a conceptual framework highlighting key research approaches and guiding principles for addressing maternal health disparities. The interconnected gears symbolize the collaborative and integrative nature of these approaches. At the core, the central gear labeled “Equal Distribution of Power, Trust, Equity” represents the foundational values that guide the integration of diverse methodologies and perspectives, emphasizing the importance of shared power, mutual respect, and equitable practices in maternal health research.

Surrounding the central gear are five distinct yet interconnected approaches:

1. Community-Based Participatory Research (CBPR): Engages communities as equal partners, centering their voices in shaping research (19).
2. Culturally Responsive and Equitable Evaluation (CREE): Emphasizes culturally relevant evaluations that respect diverse maternal health experiences (20).
3. Mixed Methods Research: Integrates qualitative and quantitative data to capture the complexity of maternal health issues.
4. Policy and Systems Research: Examines the impact of health policies and systems, identifying barriers and opportunities for reform.
5. Intersectional Analysis: Explores how overlapping intersecting identities such as race, gender, and socioeconomic status intersect to influence maternal health experiences (21).

Together, these gears represent a cohesive, multidimensional strategy for maternal health research that promotes equitable, inclusive, and contextually relevant methodologies. By integrating these approaches, the framework aims to advance understanding and drive improvements in maternal health outcomes.

## Conclusion

Reflecting on the story of Janine and Charles, we have work to do as researchers and change agents to protect birthing individuals from unjust maternal harm. It will take a multi-pronged approach to the research to move the needle in understanding psychological maternal harm. By adopting comprehensive patient-engaged research strategies that combine medical expertise with a nuanced understanding of psychological stressors through the lived experiences of birthing persons, healthcare systems can begin to dismantle systemic barriers and ensure equitable care for all birthing individuals, specifically Black birthing individuals.

## Data Availability

The original contributions presented in the study are included in the article/Supplementary Material, further inquiries can be directed to the corresponding author.
